# Relationship of Yearling Angus Bull Pulmonary Arterial Pressure Scores with Production, Maternal, and Carcass Expected Progeny Differences

**DOI:** 10.3390/ani15162430

**Published:** 2025-08-19

**Authors:** Kaylen Stearns, Hannah DelCurto-Wyffels, Sam Wyffels, Megan Van Emon, Noah G. Davis, Taylre Sitz, Tim DelCurto

**Affiliations:** 1 Department of Animal and Range Sciences, Montana State University, Bozeman, MT 59717, USA; hannah.delcurto@montana.edu (H.D.-W.); sam.wyffels@montana.edu (S.W.); megan.vanemon@montana.edu (M.V.E.); noahdavis3@montana.edu (N.G.D.); 2 Sitz Angus Ranch, Harrison, MT 59735, USA; taylreesitz@gmail.com

**Keywords:** beef cattle, expected progeny differences, pulmonary arterial pressure

## Abstract

The objectives of this study were to model the relationship of PAP scores with production, maternal, and carcass (EPDs). Bull PAP scores were collected from 12–18-month-old Angus bull from a Montana-based Angus operation (>1600 m elevation, 5400 yearlings/18-month-old bulls from 2016–2023). Bulls with greater calving ease direct had lower PAP scores (*p* < 0.01; conditional r^2^ = 1.06%; marginal r^2^ = 0.62%). Bulls with greater birth weight EPDs had greater PAP scores (*p* < 0.01; conditional r^2^ = 1.15%; marginal r^2^ = 0.73%). Pulmonary arterial pressure was greater for bulls that had greater weaning weight EPDs (*p* < 0.01; conditional r^2^ = 0.82%; marginal r^2^ = 0.16%) and for bulls that had greater yearling weight EPDs (*p* < 0.01; conditional r^2^ =0.93%; marginal r^2^ = 0.24%). Bulls with greater PAP had greater carcass weight EPDs (*p* = 0.03; conditional r^2^ = 0.69%; marginal r^2^ = 0.18%). There were no observed relationships (*p* ≥ 0.17) between bull PAP and scrotal circumference, maternal milk, ribeye area, or marbling EPDs. In summary, PAP was related to calving ease direct, birth weight, weaning weight, yearling weight, and carcass weight EPDs. However, these relationships explain very little of the variation of PAP scores.

## Simple Summary

As the cattle industry puts more emphasis and focus on heart–lung function, it is important to understand the relationship between pulmonary arterial pressure (PAP) scores with performance estimates. This study characterized the relationship of PAP scores with production, maternal, and carcass expected progeny differences (EPDs). Results showed that as the calving ease direct EPD increased, PAP scores decreased. In contrast, bulls with increased birth weight, weaning weight, yearling weight, and carcass weight EPDs had increased PAP scores. No relationship was observed between PAP scores and scrotal circumference, maternal milk, ribeye area, and marbling EPDs. By defining these relationships, beef cattle producers are provided with better insight and tools for selecting cattle for high-elevation production systems.

## 1. Introduction

An animal’s susceptibility to High-Altitude Disease (HAD), also known as Brisket Disease, High-Mountain Disease (HMD), and right-sided heart failure (RHF) is determined by its pulmonary arterial pressure (PAP) score [1]. High-Altitude Disease is described as a non-infectious disease that causes pulmonary vasoconstriction due to hypoxia [2]. This disease is characterized by clinical signs of brisket edema, lethargy, jugular vein distention, diarrhea, poor appetite, and death [3]. The only recommendation given for affected animals is to move them to lower elevation, as no treatment or cure currently exists [3]. Beef cattle located in production systems above 1500 m elevation are most impacted by HAD due to low-oxygen saturations in the atmosphere [4]. In 2012, it was estimated that 1.5 million head of cattle resided in high-elevation production systems in the United States [5]. High-Altitude Disease accounts for 3–5% of calf death loss in the United States annually [1], and results in economic losses of approximately USD 60 million, yearly [5].

Bovines have an increased susceptibility to HAD because of their pulmonary system [1,6]. During times of hypoxia, all animals will experience pulmonary vascular shunting [1] which causes persistent exposure of pulmonary vasculature to increased pressure [7]. Eventually, this increased pressure will lead to the remodeling of the pulmonary arteries caused by hypertrophy and muscularization [6,8] The hypertrophy and muscularization of the pulmonary arteries will cause loss of function in the peripheral pulmonary arteries and dilation of the right ventricle, followed by heart failure [1,6,8]. Additionally, the small, lobulated anatomical pattern of the bovine lung increases the likelihood of cattle to suffer from severe pulmonary distress and hypertension leading to loss of function [9].

Expected progeny differences (EPDs) are predictors of how future progeny are expected to perform compared to others within the data set [10]. The American Angus Association (AAA) has four different groupings of EPDs: production, maternal, management, and carcass. Beef cattle producers use bull EPDs to evaluate how bulls will fit within their operation and align with their production goals. The selection of bulls based on EPDs is a crucial part of bull selection and decision making and seedstock producers will use EPDs to market their bulls to other beef cattle producers. Selection for growth and performance within the Angus breed has trended upward for the last four decades [11]. Additionally, PAP scores are also used as a marketing tool by seedstock producers to market their cattle particularly in high-elevation systems to those beef cattle operations who put an emphasis on heart–lung function in their selection criteria [12].

The AAA PAP EPD was developed and released in 2019 by the AAA, Angus Genetics Inc. (AGI), and Colorado State University [13]. This EPD assists in estimating an animal’s “survivability” in high-elevation environments [14]. Just as the PAP score, the EPD is also expressed in mmHg and a lower score is more favorable [13]. However, the AAA emphasizes that the PAP EPD does not replace a PAP test and there are several other environmental factors that may impact an animal’s survivability in high-elevation beef cattle production [13,15]. However, producers are able to use this EPD as a guide to make informed decisions when selecting and have an increased knowledge on how expected progeny should perform in high-elevation environments [15]. Increased recognition of the importance of heart–lung function led to the establishment of the PAP EPD, as well as increased interest in PAP measurements.

There is limited research conducted relating PAP measurements to production, maternal, and carcass EPDs. The objectives of this study were to model the relationship of PAP with production, maternal, and carcass EPDs. Increased knowledge of PAP scores and the relationship between EPDs and PAP scores can allow for a better understanding of selection goals in beef cattle.

## 2. Materials and Methods

We collaborated with a Montana-based Angus operation to gather data on 5406 head of 12- to 18-month-old purebred Angus bulls which were sold in their annual production sales from 2016 to 2023. Bulls continuously resided in elevations greater than 1600 m. It is important to note that this operation has two separate locations that function separately from each other, meaning they have different management plans and operational goals.

### 2.1. Operation A

Bulls from operation A are sold in the December production sale which will be referred to as the fall sale and are PAP tested at 18 months of age. Bulls from operation A are weaned in a pasture setting in the fall, then sent to the ranch feedlot for the winter. In the spring, bulls are turned out on forest allotments (≈2590 m elevation) to graze. They return back to the feedlot (≈1600 m) in the fall where all sale bulls are PAP tested following their return from elevation. While at the feedlot, they receive a light growing ration until sale.

### 2.2. Operation B

Bulls from operation B are sold in the March production sale which will be referred to as the spring sale and are PAP tested at 12 months of age. Bulls from operation B are weaned into a feedlot (≈1600 m elevation) in early September. The bulls are sorted into groups by weight and started on a high-roughage diet. From there, concentrates of corn and dried distillers grains are gradually added into the ration. Sale bulls are PAP tested in the middle of December.

### 2.3. Pulmonary Arterial Pressure Test

Through a right-heart catheterization procedure, a PAP test measures the resistance of blood flow through the lungs [16]. Measurement of PAP has been routinely performed by a licensed veterinarian at beef cattle operations at high elevations since the 1980s. Similar to human blood pressure, PAP scores are measured in millimeters of mercury (mmHg) [1]. Scores range from 30 mmHg to greater than 50 mmHg [1] and are influenced by a variety of factors, including age, sex, genetics, and environmental conditions [1,17,18,19,20]. Higher scores indicate high susceptibility to HAD, therefore lower scores are more desirable [1].

### 2.4. Data Collected

Data gathered included identification number, sale, sale year, birthdate, PAP score, along with EPDs for calving ease direct (CED), birth weight (BW), weaning weight (WW), yearling weight (YW), scrotal circumference (SC), maternal milk (Milk), carcass weight (CW), marbling (Marb), and ribeye area (RE; Table 1). The provided genomically enhanced EPDs are reflective of bulls’ calculated estimates assigned by the AAA at time of sale and as published in the producer’s bull sale catalogs. This is the second paper in a two-paper series through a collaboration with Sitz Angus who uses PAP testing as a standard operation procedure for all bulls marketed through their annual production sales [21].

### 2.5. Statistical Analysis

Generalized linear mixed models were used to determine the relationship between PAP scores with production, maternal, and carcass EPDs. Candidate models were developed reflecting hypothesized relationships between PAP and the maternal, production, and carcass EPDs as either linear, asymptotic, or quadratic in nature [22], and year as a random intercept. Akaike’s Information Criterion adjusted for small sample sizes (AICc) was used to evaluate support for competing models (Table 2; “AICcmodavg” package for R) [23]. Once the nature of the relationship was determined for each EPD of interest, candidate model sets using generalized linear mixed models were then developed reflecting multiple hypotheses on the relationship between each of the EPDs including fall versus spring sale and its associated interactions with each EPD of interest on bull PAP scores using the packages “lme4” and “lmerTest” (packages for R) [24,25]. Spring versus fall sale was treated as a categorical fixed effect to account for age and management effects between operations. Year was treated as a random intercept. Akaike’s Information Criterion adjusted for small sample sizes (AICc) was used to evaluate support for competing (Table 3; “AICcmodavg” package for R) [23]. We excluded models with ΔAICc ≤ 2 that differed from the top model by a single parameter if confidence intervals of a parameter estimates overlapped 0 (i.e., were non-informative) [26]. Model averaged estimates of beta-coefficients were used when multiple models were supported (“MuMin” package for R) [27]. Model fit was evaluated by using marginal and conditional r^2^ [28]. All models and relationships were performed and evaluated in R [29]. Significance was determined at an alpha of <0.05 with a tendency discussed at an alpha > 0.05 and <0.10.

## 3. Results

There were no significant relationships between bull PAP and SC EPD (Table 3; *p* = 0.28; fall sale: β = −0.20 ± 0.24; spring sale: β = −0.77 ± 0.42), Milk EPD (*p* = 0.51; β = 0.28 ± 0.45), RE EPD (*p* = 0.17; fall sale: β = −0.47 ± 0.58; spring sale: β = −1.07 ± 0.77), and Marb EPD (*p* = 0.21; fall sale: β = −0.17 ± 0.41; spring sale: β = −0.74 ± 0.59). These models may be noninformative as the confidence intervals of the effect overlap 0.

**Figure 1:**
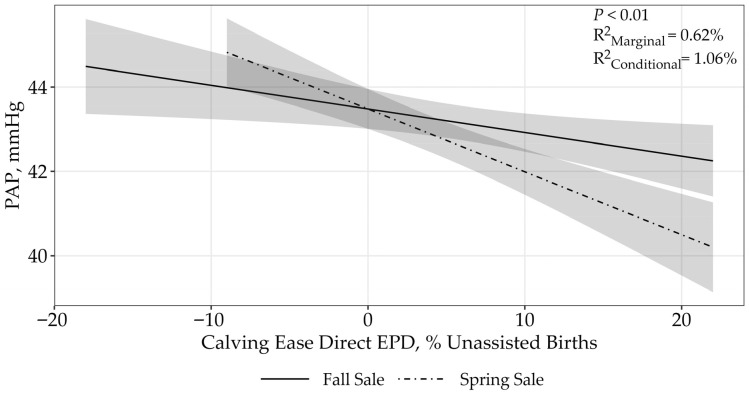
Predicted relationship (±95% CI represented in the shaded area) between PAP scores and CED EPD. This relationship was best represented by a log-transformed model. Calving ease direct is expressed as a difference percentage of unassisted births, with a higher value indicating greater calving ease in first-calf heifers. The solid line represents the fall sale hosted by operation A, while the dashed line represents the spring sale hosted by operation B.

The CED EPD displayed an asymptotic relationship (*p* < 0.01) to PAP, with candidate models containing CED EPD and sale receiving 92% of the relative support (Table 3; *p* < 0.01, fall sale: β = −0.06 ± 0.02; spring sale: β = −0.15 ± 0.03; Figure 1). These findings suggest bulls with greater CED had lower PAP scores. The top model containing all the supported variables had a conditional r^2^ = 1.06% and a marginal r^2^ = 0.62%, suggesting that the CED EPD and sale only accounted for 0.62% of the variation associated with PAP.

**Figure 2:**
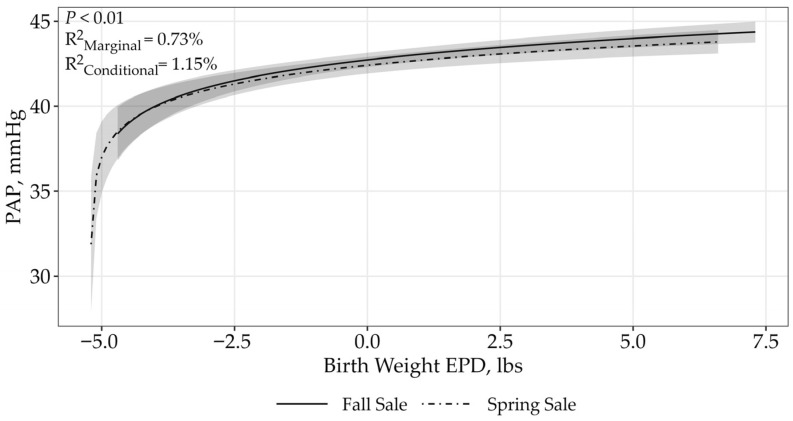
Predicted relationship (±95% CI represented in the shaded area) between PAP scores and BW EPD. This relationship was best represented by a log-transformed model. The BW EPD is expressed in pounds and predicts the sire’s ability to transmit birth weight to his progeny. The solid line represents the fall sale hosted by operation A, while the dashed line represents the spring sale hosted by operation B.

For BW EPD, there was model uncertainty (ΔAIC_c_ < 2; Table 3). However, the BW EPD by sale interaction displayed an asymptotic relationship to PAP scores (*p* < 0.01; fall sale: β = 1.87 ± 0.32; spring sale: β = 1.68 ± 0.32; Figure 2). Bulls with higher BW EPDs had higher PAP scores. The top model containing all supported variables had a conditional r^2^ of 1.15% and a marginal r^2^ of 0.73%, suggesting that birth weight and sale only accounted for 0.73% of the variation associated with PAP.

**Figure 3:**
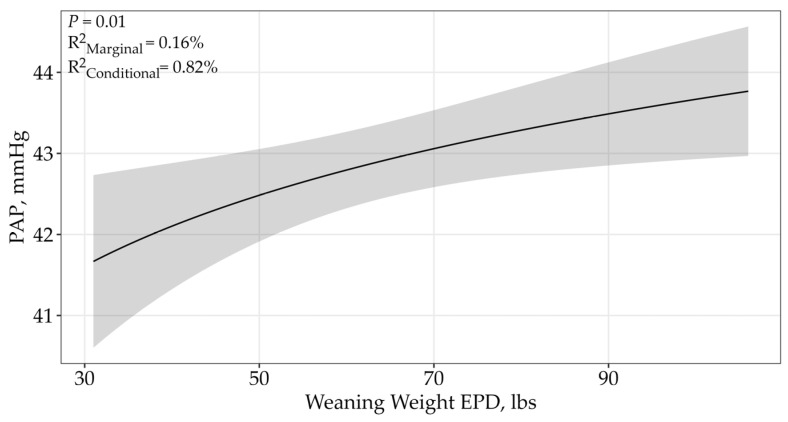
Predicted relationship (±95% CI represented in the shaded area) between PAP scores and WW EPD. This relationship was best represented by a log-transformed model. The WW EPD is expressed in pounds and predicts the sire’s ability to transmit weaning weight to his progeny. There was no difference between the two sales.

A single top model containing the WW EPD received 78% of support among candidate models indicating there was no difference between sales (Table 3; *p* = 0.01; β = 1.71 ± 0.67; Figure 3. Pulmonary arterial pressure scores increased as the WW EPD increased. The top model containing all the supported variables had a conditional r^2^ of 0.82% and a marginal r^2^ of 0.16%, therefore suggesting that the WW EPD only accounts for 0.16% of the variation in PAP scores.

**Figure 4:**
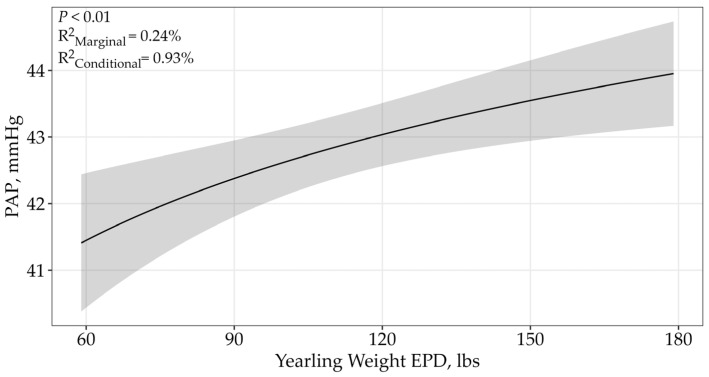
Predicted relationship (±95% CI represented in the shaded area) between PAP scores and YW EPD. This relationship was best represented by a log-transformed model. The YW EPD is expressed in pounds and predicts the sire’s ability to transmit yearling weight to his progeny. There was no difference between the two sales.

The YW EPD displayed an asymptotic relationship (*p* < 0.01) with bull PAP, and the single top model received 77% of the support among the candidate model, suggesting there was no difference between sales. Pulmonary arterial pressure scores increased as YW EPDs increased (Table 3; β = 2.29 ± 0.71; Figure 4). The top model containing all supported variables had a conditional r^2^ of 0.93% and a marginal r^2^ of 0.24%.

**Figure 5:**
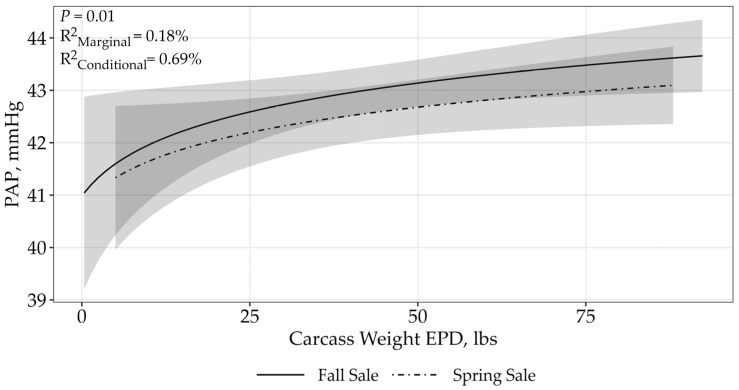
Predicted relationship (±95% CI represented in the shaded area) between PAP scores and CW EPD. This relationship was best represented by a log-transformed model. The CW EPD is expressed in pounds and the difference in hot carcass weight of a sire’s progeny compared to the progeny of other sires. The solid line represents the fall sale hosted by operation A, while the dashed line represents the spring sale hosted by operation B.

For the CW EPD, there was model uncertainty (ΔAICc < 2; Table 3). However, an asymptotic relationship best described the relationship between PAP and the CW EPD (*p* = 0.03; fall sale: β = 0.77 ± 0.35; spring sale: β = 0.65 ± 0.35; Figure 5), where PAP increased as the CW EPD increased. The top model containing all supported variables had conditional r^2^ of 0.69% and marginal r^2^ of 0.17%.

## 4. Discussion

Our study uses Angus seedstock performance-driven bulls to assess the relationship between production, maternal, and carcass EPDs with PAP scores. Findings from this study aid in characterizing the relationship between production, maternal, and carcass EPDs with PAP scores. While there have been studies establishing a relationship between PAP scores with actual performance, such as birth weight and yearling weight, the results have been conflicting [16,21,30]. In addition, information evaluating the relationship of EPDs with PAP scores is limited. Therefore, in this study, we chose to evaluate PAP’s relationship to specific production, maternal, and carcass EPDs.

The SC EPD is expressed in centimeters and is a predictor of the difference in transmitting ability for scrotal size compared to that of other sires. The Milk EPD is a predictor of a sire’s genetic merit for milk and mothering ability as expressed in his daughter compared to daughters of other sires. In our study, we observed no relationship between the SC EPD and the Milk EPD to PAP scores.

The Marb EPD is expressed as a fraction of the differences in United State Department of Agriculture marbling score of a sire’s progeny compared to progeny of other sires [10]. This study found no significance between the Marb EPD and PAP scores. In contrast, a previous study showed low genetic correlation (<0.20) between PAP scores and ultrasound values for backfat, rump fat, and intramuscular fat [11].

The RE EPD is expressed in square inches and is a predictor of the differences in the ribeye area of a sire’s progeny compared to progeny of other sires [10]. No relationship was observed between RE EPD and PAP scores in this study. However, a previous study yielded conflicting results. Pauling et al. observed a moderate positive genetic correlation (0.25 ± 0.12) between PAP scores and ultrasound ribeye area values [11]. The results from the previous study may indicate that single trait selection for ribeye may cause an increase in PAP scores [11].

Calving ease direct is expressed as a difference in percentage of unassisted births, with a higher value indicating greater calving ease in first-calf heifers [10]. It predicts the average difference in ease with which a sire’s calves will be born when he is bred to first-calf heifers [10]. Looking into the relationship between CED and PAP scores, the current study found that as CED increases, the PAP measurements decrease.

The BW EPD is expressed in pounds and predicts the sire’s ability to transmit birth weight to his progeny compared to that of other sires [10]. Similarly, our candidate model indicated that as the BW EPD increased, PAP measurements also increased. Previous research demonstrated that birth weight was positively and moderately genetically correlated to PAP [16]. When comparing the relationship of PAP scores with actual birth weight, it showed that as birth weight increased, PAP scores did as well [16,21].

The WW EPD is expressed in pounds and predicts the sire’s ability to transmit weaning growth to his progeny compared to that of other sires [10]. The relationship between WW EPD and PAP scores suggested that as the WW EPD increased, so did PAP scores. There have been conflicting results in previous research analyzing the relationship of PAP to WW. One study observed no significant phenotypic correlation between actual weaning weight and PAP measurements [21], while a different study indicated that the phenotypic correlation showed to be positive but low (0.02 ± 0.03) [16]. Genotypic correlations were weak to moderate relationships between PAP scores and weaning weights (0.22 ± 0.08; 0.50 ± 0.18) [16,30].

The YW EPD is expressed in pounds and predicts the sire’s ability to transmit yearling growth to his progeny compared to that of other sires [10]. Our study suggested that as YW EPD increased, PAP scores increased. This result conflicted with phenotypic correlations which investigated the relationship between PAP scores and actual yearling weight, and showed that as actual yearling weight increased, PAP measurements decreased [21]. Previous genotypic correlations have yielded conflicting results. Two previous genotypic correlations indicated that there is a weak genetic correlation between PAP and YW [11,30]. However, an earlier study concluded there was a strong, negative relationship between actual yearling weights and PAP measurements [31].

Finally, the CW EPD, expressed in pounds, is a predictor of the difference in hot carcass weight of a sire’s progeny compared to the progeny of other sires. Our results concluded that as the CW EPD increased, PAP also increased. A previous regression analysis of actual hot carcass weight and PAP suggested there was a negative relationship [14]. This indicated that on average, high PAP steers (>49 mmHg) had a 15 kg decrease in carcass weight compared to those steers who were grouped into low and moderate PAP categories [14].

While PAP was found to be associated with several performance, maternal, and carcass EPDs, the amount of variation explained by these relationships is less than 1%, individually. Pulmonary arterial pressure is a means to estimate heart–lung function, and, in turn, heart–lung function is dependent on many factors, both genetic and environmental. This can be illustrated in our study by evaluating the difference between conditional and marginal r^2^, which represents the amount of variation explained by the year of testing, one environmental variable that often explains as much or more of the variation of PAP scores as the EPDs. Similarly, conflicting results from previous studies may be due to environmental, production, and management differences.

Three of the EPDs, CED, BW, and CW showed a difference between sales. This can potentially be attributed to several factors including age at time of testing, management strategies, and environmental differences. Operation A PAP tests their bulls at 18 months of age compared to operation B which tests their bulls at 12 months of age. This 6-month age gap could account for this differences in sale [1,12]. Additionally, differing management strategies such as nutritional program, breeds, and genetics can cause differences in PAP scores and heart–lung function [1,12]. Environmental factors such as elevation and temperature at the time of testing can also influence PAP scores [1,12]. All these factors may contribute to the differences within sales.

However, our results suggest that selecting for greater growth and performance using EPDs can result in greater PAP, whereas those bulls that experience greater actual growth and performance have lower PAP scores [21]. This demonstrates the importance of keeping heart–lung function in mind when selecting cattle as results indicate those cattle with improved heart–lung function will capitalize on their genetic potential for growth and performance.

Our study characterized the relationship between PAP and production, maternal, and carcass EPDs in yearling Angus bulls; however, future studies are warranted to address current study limitations including the inability to repeat individual bull PAP tests to ensure repeatability and accuracy. Additionally, environmental conditions vary at each operation and each year. Additional research regarding genetic and environmental variations is warranted to further understand the impact of management strategies and genetic selection regarding heart–lung function. Furthermore, research and development to find a less invasive and more accessible measurement of PAP is needed to continue to improve heart–lung function in beef cattle.

## 5. Conclusions

Heart–lung function continues to be a point of emphasis in the beef cattle sector. By determining relationships between PAP scores and EPDs that are points of selection for producers, we can better understand how these relationships can impact beef production in high-elevation systems. It is important to keep heart–lung function in mind when selecting for growth and performance. This research showed significant relationships between PAP and the CED, BW, WW, YW, and CW EPDs. However, these relationships explain very little of the variation in PAP scores. Still, findings from this study can help better understand the importance of PAP in relation to growth and performance in high-elevation production systems when determining an operation’s goals for management. This research provides insight on the importance of selecting for heart–lung function to maximize growth and performance of beef cattle which is critical as we move toward selecting for increased growth and carcass weights.

## Figures and Tables

**Table 1:** Expected progeny differences evaluated within this study with the minimum, mean ± standard deviation (SD), and maximum. Groupings and abbreviations are according to the AAA ^1^. The actual PAP score was used and not the EPD.

Name	Minimum	Mean ± SD	Maximum
PAP	25	43.00 ± 7.40	1250.00
**Production EPDs**			
Calving Ease Direct (CED)	−13.00	6.75 ± 4.77	22.00
Birth Weight (BW)	−4.70	1.13 ± 1.75	7.30
Weaning Weight (WW)	31.00	66.95 ± 11.03	106.00
Yearling Weight (YW)	59.00	117.33 ± 17.89	179.00
Scrotal Circumference (SC)	−1.09	1.08 ± 0.56	2.89
**Maternal EPDs**			
Maternal Milk (Milk)	0.29	27.48 ± 5.82	48
**Carcass EPDs**			
Carcass Weight (CW)	0.35	47.62 ± 12.17	93.00
Marbling (Marb)	−0.41	0.49 ± 0.93	51.00
Ribeye Area (RE)	−0.56	0.58 ± 1.16	65.00

^1^ American Angus Association.

**Table 2:** Model selection for evaluating the nature (linear, asymptotic, or quadratic) of the relationship between PAP and production, maternal, and carcass EPDs on 12–18-month-old Angus bulls. Top models reflecting the nature of the relationship between PAP and EPDs are bolded.

Model ^1^	K ^2^	AICc ^3^	ΔAICc ^4^	W_i_^5^
Calving Ease Direct				
**ln(Calving Ease Direct)**	**4**	**33,842.86**	**0**	**0.52**
**Calving Ease Direct**	**4**	**33,843.04**	**0.18**	**0.48**
(Calving Ease Direct)^2^	5	33,852.78	9.92	0
Birth Weight				
**ln(Birth Weight)**	**4**	**33,791.67**	**0**	**0.76**
Birth Weight	4	33,794.03	2.36	0.23
(Birth Weight)^2^	5	33,801.02	9.35	0.01
Weaning Weight				
**ln(Weaning Weight)**	**4**	**33,817.08**	**0**	**0.99**
Weaning Weight	4	33,825.98	8.91	0.01
(Weaning Weight)^2^	5	33,839.94	22.86	0
Yearling Weight				
**ln(Yearling Weight)**	**4**	**33,776.60**	**0**	**0.99**
Yearling Weight	4	33,786.75	10.16	0.01
(Yearling Weight)^2^	5	33,802.34	25.74	0
Maternal Milk				
**ln(Maternal Milk)**	**4**	**33,787.26**	**0**	**0.97**
Maternal Milk	4	33,793.92	6.67	0.03
(Maternal Milk)^2^	5	33,895.85	18.60	0
Scrotal Circumference				
**ln(Scrotal Circumference)**	**4**	**22,413.49**	**0**	**0.63**
**Scrotal Circumference**	**4**	**22,415.05**	**1.56**	**0.29**
(Scrotal Circumference)^2^	5	22,417.43	3.94	0
Ribeye Area				
**ln(Ribeye Area)**	**4**	**33,800.94**	**0**	**0.91**
Ribeye Area	4	33,805.60	4.66	0.09
(Ribeye Area)^2^	5	33,814.41	13.46	0
Marbling				
**ln(Marbling)**	**4**	**33,808.49**	**0**	**0.77**
Marbling	4	33,810.94	2.45	0.23
(Marbling)^2^	5	33,819.91	11.42	0
Carcass Weight				
**ln(Carcass Weight)**	**4**	**33,805.00**	**0**	**0.97**
Carcass Weight	4	33,812.30	7.31	0.03
(Carcass Weight)^2^	5	33,827.75	22.25	0

^1^ Year is used as a random variable in all models, ln = Natural Logarithmic Model, (x)^2^ = Quadratic Model ^2^ K is the number of parameters. ^3^ Akaike’s Information Criterion adjusted for small sample size. ^4^ Difference in Akaike’s Information Criterion adjusted for small sample size compared to the best model. ^5^ Akaike weight.

**Table 3:** Model selection for evaluating the effects of production, maternal, and carcass EPDS, fall versus spring sale, and associated interactions on 12- to 18-month-old purebred Angus bull PAP scores. Top models for each EPD are bolded.

Model ^1^	K ^2^	AICc ^3^	ΔAICc ^4^	W_i_ ^5^	r^2^m ^6^	r^2^c ^7^
Calving Ease Direct						
**ln(Calving Ease Direct) × sale**	**5**	**33,838.18**	**0**	**0.92**	**0.62%**	**1.06%**
ln(Calving Ease Direct)	4	33,843.04	4.86	0.08	0.36%	0.81%
Constant (null)	3	33,858.87	20.69	0	0%	0.50%
Birth Weight						
**ln(Birth Weight)**	**4**	**33,791.67**	**0**	**0.73**	**0.67%**	**1.10%**
**ln(Birth Weight) × sale**	**5**	**33,793.65**	**1.98**	**0.27**	**0.73%**	**1.15%**
Constant (null)	3	33,858.87	67.21	0	0%	0.50%
Weaning Weight						
**ln(Weaning Weight)**	**4**	**33,817.08**	**0**	**0.78**	**0.16%**	**0.82%**
ln(Weaning Weight) × sale	5	33,819.66	2.59	0.22	0.21%	0.80%
Constant (null)	3	33,858.87	41.80	0	0%	0.50%
Yearling Weight						
**ln(Yearling Weight)**	**4**	**33,776.60**	**0**	**0.77**	**0.24%**	**0.92%**
ln(Yearling Weight) × sale	5	33,778.96	2.36	0.23	0.30%	0.92%
Constant (null)	3	33,858.87	82.28	0	0%	0.50%
Maternal Milk						
**ln(Maternal Milk)**	**4**	**33,787.26**	**0**	**0.64**	**0.008%**	**0.49%**
**ln(Maternal Milk) × sale**	**5**	**33,788.44**	**1.18**	**0.36**	**0.10%**	**0.56%**
Constant (null)	3	33,858.87	71.62	0	0%	0.50%
Scrotal Circumference						
**ln(Scrotal Circumference) × sale**	**5**	**22,415.02**	**0**	**0.50**	**0.12%**	**1.50%**
**ln(Scrotal Circumference)**	**4**	**22,415.05**	**0.03**	**0.50**	**0.04%**	**1.30%**
Constant (null)	3	33,858.87	11,443.86	0	0%	0.50%
Ribeye Area						
**ln(Ribeye Area) × sale**	**5**	**33,800.85**	**0**	**0.51**	**0.05%**	**0.53%**
**ln(Ribeye Area)**	**4**	**33,800.94**	**0.10**	**0.49**	**0.04%**	**0.53%**
Constant (null)	3	33,858.87	58.03	0	0%	0.50%
Marbling						
**ln(Marbling)**	**4**	**33,808.49**	**0**	**0.53**	**0.02%**	**0.51%**
**ln(Marbling) × sale**	**5**	**33,808.71**	**0.23**	**0.47**	**0.04%**	**0.51%**
Constant (null)	3	33,858.87	50.39	0	0%	0.50%
Carcass Weight						
**ln(Carcass Weight)**	**4**	**33,805.00**	**0**	**0.72**	**0.10%**	**0.68%**
**ln(Carcass Weight) × sale**	**5**	**33,806.88**	**1.89**	**0.28**	**0.17%**	**0.69%**
Constant (null)	3	33,858.87	53.88	0	0%	0.50%

^1^ Year is used as a random variable in all models. ln = Natural Logarithmic Model. ^2^ K is the number of parameters. ^3^ Akaike’s Information Criterion adjusted for small sample size. ^4^ Difference in Akaike’s Information Criterion adjusted for small sample size compared to the best model. ^5^ Akaike weight. ^6^ Marginal r^2^. ^7^ Conditional r^2^.

## Data Availability

Data are owned by Sitz Angus Ranch. Access to data can only be obtained by permission from the Sitz Angus Ranch.

## Abbreviations

The following abbreviations are used in this manuscript:

HAD	High-Altitude Disease
HMD	High-Mountain Disease
RHF	Right-sided heart failure
EPD	Expected Progeny Difference
AAA	American Angus Association
AGI	Angus Genetics Inc.
CED	Calving Ease Direct
BW	Birth Weight
WW	Weaning Weight
YW	Yearling Weight
SC	Scrotal Circumference
Milk	Maternal Milk
CW	Carcass Weight
Marb	Marbling
RE	Ribeye Area
AICc	Akaike’s Information Criterion
